# Enigma of Bowel Angina: Unraveling Celiac Trunk Stenosis

**DOI:** 10.7759/cureus.40258

**Published:** 2023-06-11

**Authors:** Abeer Qasim, Venkata Sri Ramani Peesapati, Jay Patel, Joshua Davidson

**Affiliations:** 1 Internal Medicine, BronxCare Health System, New York, USA; 2 Pulmonary and Critical Care Medicine, BronxCare Health System, New York, USA

**Keywords:** median arcuate ligament syndrome, dunbar syndrome, celiac axis syndrome, bowel angina caused by celiac trunk stenosis, celiac artery stenosis

## Abstract

The celiac axis is commonly involved in conditions that cause the narrowing or blockage of a celiac artery atherosclerosis and other vascular disease. Celiac artery compression syndrome is defined as chronic abdominal pain occurring because of compression of the celiac artery commonly in middle-aged (40 to 60 years) females. Various etiologies include atherosclerosis of mesenteries vessels, pancreatitis, median arcuate ligament syndrome, and tumor invasion. It is an uncommon condition, and symptoms include post-prandial abdominal pain mostly in the epigastrium, weight loss, nausea, diarrhea, anorexia, and bloating. Patients are asymptomatic for a prolonged duration due to collateral blood supply to the bowel from the patent superior mesenteric and inferior mesenteric arteries. We present a case of a 67-year-old female who initially presented with signs and symptoms suggestive of small bowel obstruction, however, due to persistent abdominal pain, she underwent a CT scan suggestive of severe celiac trunk stenosis causing abdominal angina. The patient was managed conservatively and responded well with close follow-up.

## Introduction

Celiac trunk stenosis is a condition caused by constriction of the celiac trunk, a major branch of the abdominal aorta that supplies blood to vital abdominal organs, including the liver, spleen, and intestines. Risk factors include age, smoking, hypertension, diabetes, and hyperlipidemia. Incidence ranges from 12.5-24% in Caucasians. According to Sakorafas et al., the etiology of celiac trunk stenosis is classified as intrinsic (due to atherosclerosis), extrinsic due to median arcuate ligament syndrome, and other causes like malignancy [1]. Diagnosis of the syndrome includes ultrasound, CT angiogram, multi-detector CT scanners, and magnetic resonance angiography along with suggestive clinical features. In cases with high suspicion, ultrasound can also be used as a screening test [2]. Treatment involves conservative as well as invasive management with the surgical division of the median arcuate ligament, celiac plexus, and celiac ganglia [3]. Our patient presented with features suggestive of severe celiac trunk stenosis.

## Case presentation

A 67-year-old female with a history of congestive heart failure, chronic obstructive pulmonary disease, human immunodeficiency virus (HIV), hyperlipidemia, hypertension, seizures, asthma, recurrent Clostridium difficile (C. diff), and ulcerative colitis (UC) who initially presented to the emergency department (ED) with complaints of lower abdominal pain associated with nausea and multiple episodes of non-bilious, non-bloody emesis. The patient stated these symptoms began one day prior to arrival and denied any previous similar episodes. She also had diarrhea, with her most recent bout occurring in the ED earlier that morning; however, she denied any fevers, chills, hematochezia, recent antibiotic use, and travel history. The patient had a surgical history of tubal ligation, cholecystectomy, and appendectomy. Family history was relevant for Crohn’s disease in her son. The patient also had a 40-pack-year smoking history, denied current or recent alcohol use, and her urine toxicology was negative. Her home medications are metformin, keppra, lasix, nifedipine, Descovy, Tivicay, montelukast, labetalol, atorvastatin, insulin Lantus, and bronchodilators.

The patient was hemodynamically stable on admission with a heart rate of 78 beats per minute, blood pressure of 116/56 mmHg, temperature of 97.8 degrees Fahrenheit, and respiratory rate of 18. On initial physical examination, the patient had a soft abdomen, with tenderness in the right upper quadrant and bilateral lower quadrants on light palpation. Bowel sounds were hypoactive. The patient was admitted to the hospital for additional evaluation, given that patient was having persistent abdominal pain. Initial labs were significant for hypomagnesemia of (1.4 mg/dL) and lactic acidosis (2.3 mmoles/L).

**Figure 1:**
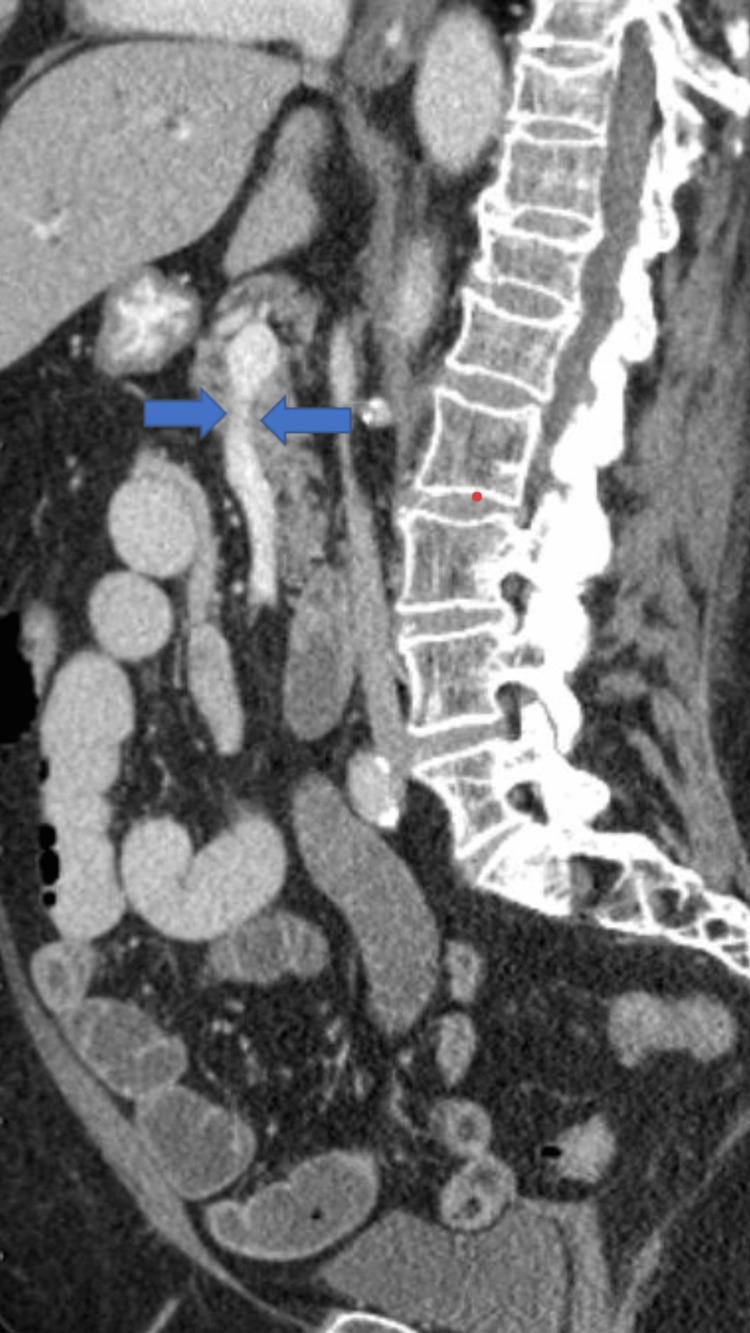
Sagittal view of CT scan of the abdomen with contrast showing celiac artery stenosis (blue arrows)

**Figure 2:**
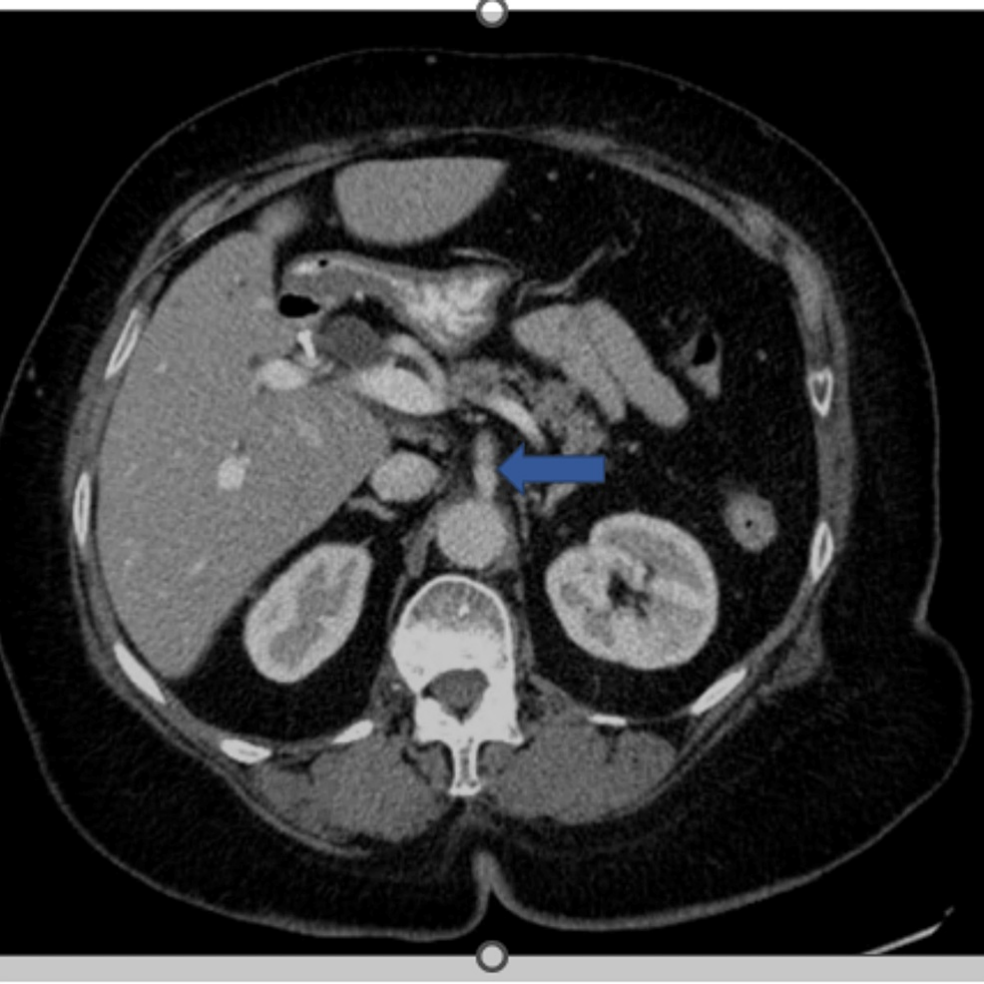
Axial view of CT abdomen and pelvis with contrast showing celiac trunk stenosis (blue arrows)

Due to persistent abdominal pain, the patient underwent a CT scan of the abdomen and pelvis with contrast, which revealed small bowel obstruction (SBO) and severe stenosis for the celiac trunk (Figures 1, 2).

**Figure 3:**
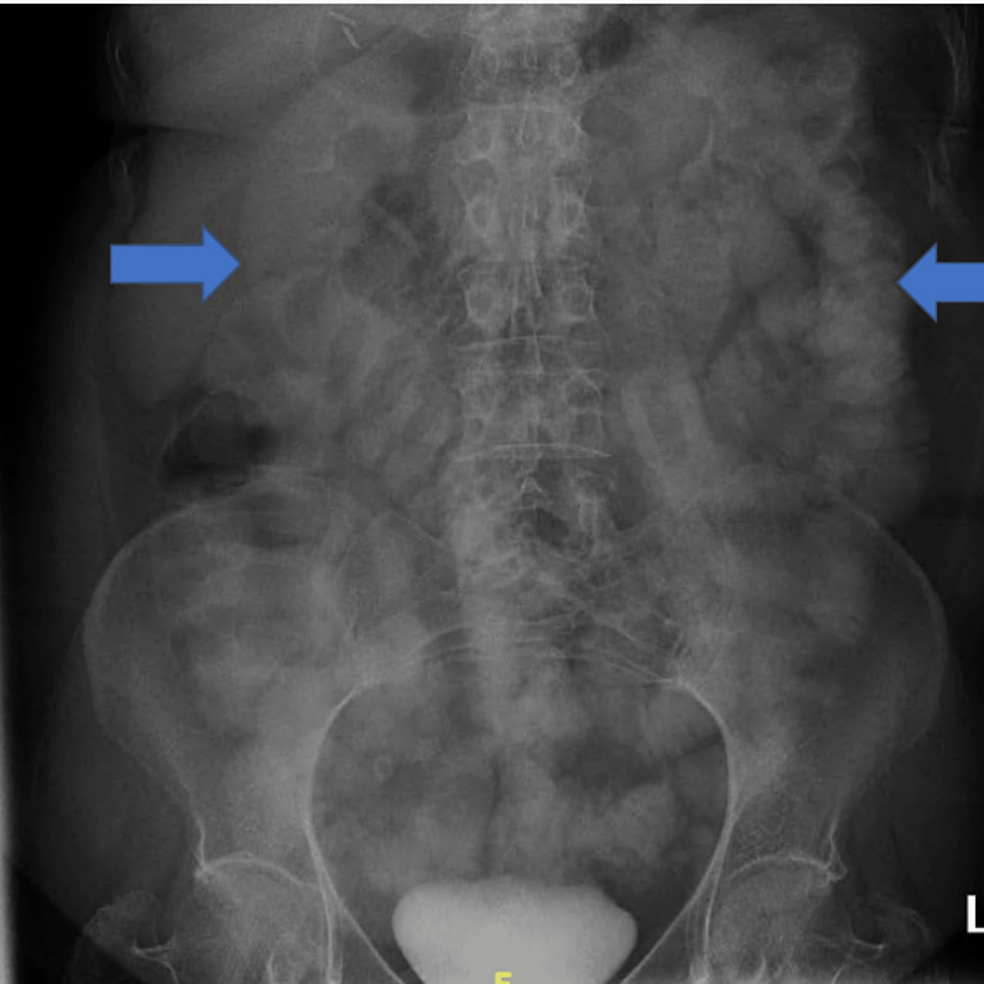
X-ray of the abdomen with gastrograffin There is contrast throughout the small and large bowel with some dilated small bowel loops, the largest measuring 3.5 cm. No free air or abnormal calcifications are seen.

Surgery was consulted due to SBO; however, on physical examination, there was no evidence of abdominal distension. Surgery recommended an X-ray abdomen with gastrograffin challenge, which revealed partial or resolved small bowel obstruction (Figure 3).

On serial abdominal examinations, the patient had a soft abdomen with tenderness to deep palpation of the bilateral lower quadrants; there was no active guarding or rebound tenderness. Bowel sounds were normoactive. Additional testing was done for C. diff, repeat CD4 count, and HIV viral load, which were all negative (Table 1).

**Table 1:** Laboratory parameters results GDH: glutamate dehydrogenase; PCR: polymerase chain reaction

Laboratory parameters	Results	Reference range and units
Hemoglobin	12.2	12–16 g/dL
Hematocrit	37.2	42–51%
White blood cell count	8.4	4.8–10.8 K/µL
Platelets	404	150–400 K/µL
Sodium	146	135–145 mEq/L
Potassium	3.7	3.5–5.0 mEq/L
Blood urea nitrogen	13.0	6–20 mg/dL
Creatinine	0.7	0.5–1.5 mg/dL
Chloride	101	98–108 mEq/L
Calcium	8.8	8.5–10.5 mg/dL
Magnesium	1.7	1.5–2.7 mg/dL
Phosphorus	2.6	2.5–4.5 mg/dL
Aspartate aminotransferase	23	9–33 units/L
Alkaline phosphatase	48	43–160 units/L
Stool GDH antigen	Not Detected	Not Detected
Absolute CD4 count	673	490-1740 cells/uL
HIV RNA QN PCR copies	29	Not Detected
Lactic acid	2.3 mmoles/L	0.5-1.6 mmoles/L

The gastrointestinal (GI) team was consulted during the patient's hospital course. Nevertheless, recent colonoscopy records revealed scattered small and large-mouthed diverticula in the sigmoid colon. The GI service recommended no further intervention. Given the patient’s presentation, she was started on intravenous fluids, anti-emetics, and a bowel regimen. Despite the resolution of her SBO, the patient had episodic abdominal pain. Due to the patient’s age, social history, and CT scan findings, her symptoms were highly suggestive of celiac artery stenosis. The patient was planned for additional evaluation, including a CT angiogram, but she wanted to follow up as an outpatient as she had symptomatic relief. Moreover, her laboratory findings, including lactic acidosis and electrolyte imbalances, were improved,

## Discussion

The celiac trunk, also known as Haller’s tripod, supplies blood to the majority of the upper gastrointestinal tract, including the stomach, duodenum, pancreas, liver, and spleen. Celiac artery stenosis (CAS) is a serious yet subtle clinical problem. The trunk divides into three significant arteries, which include the common hepatic artery, splenic artery, and left gastric artery. Celiac trunk anatomic variations can predispose patients to develop CAS, and one such variation is the hepatic-splenic-mesenteric trunk where the branches directly originate from the abdominal aorta. CAS etiology can be organic or functional. Organic causes include atherosclerosis, fibromuscular dysplasia, and polyarthritis nodosa [4]. Many patients with organic CAS are asymptomatic due to the collateral circulation between the celiac trunk and mesenteric arteries [5]. The functional cause of CAS is due to median arcuate ligament syndrome (MALS) where extrinsic compression by the median arcuate ligament causes celiac trunk stenosis.

MALS, also known as Dunbar syndrome, is a diagnosis of exclusion and often presents with a triad of persistent abdominal pain, weight loss, and inflammatory bowel disorder (IBD) symptoms [6]. Hence, it can mimic symptoms of life-threatening pathologies, including Crohn’s disease and ulcerative colitis. MALS is a reversible condition that significantly improves the patient’s quality of life after treatment, however, due to its non-specific presentation, it is a challenging and under-diagnosed condition [7,8].

Diagnosis is dependent on the patient’s duration of symptoms, medical history, clinical picture, and radiologic evidence. CT abdominal angiography remains the gold standard technique for diagnosing CAS due to organic causes, whereas mesenteric duplex ultrasound can aid in the diagnosis of MALS. Turbulent blood flow with increased blood flow velocity > 200 cm/s during expiration with normalized waveforms on inspiration confirms the diagnosis of celiac artery compression on ultrasound [7]. Endovascular revascularization, celiac plexus block, and laparoscopic surgical decompression of the arcuate ligament are effective interventions for CAS treatment. Although endovascular revascularization is associated with less mortality and morbidity, open revascularization provides extended symptomatic relief [9]. Complications of endovascular intervention include stent damage, restenosis, and compression of the stent if MALS is the primary culprit [10].

As with our patient, celiac trunk stenosis manifests as non-specific abdominal symptoms, especially in the elderly. CAS remains an overlooked diagnosis to date, and patients undergo several investigations with unnecessary treatments before a definitive diagnosis is made. Clinicians must always be mindful of celiac artery compression as a differential diagnosis of atypical abdominal pain as prompt recognition and treatment can provide immediate relief to the patient.

## Conclusions

In summary, bowel angina due to celiac axis trunk stenosis leads to inadequate blood supply to abdominal organs, resulting in ischemia and subsequent symptoms. Atherosclerosis and MALS are the two main causes of CAS. Early diagnosis and management are crucial to prevent complications and improve patients’ symptoms. Treatment depends on the severity of stenosis and the duration of the illness. Mild and asymptomatic patients can be monitored by controlling risk factors. Severe cases require urgent surgical intervention, including angioplasty with or without stent placement and decompression of the median arcuate ligament. Celiac artery stenosis, often misdiagnosed in the elderly population, is an unusual yet easily reversible cause of abdominal pain.
